# Solitude Misremembered: Recalled Loneliness and Happiness in Solitude Are Shaped by Age, Culture, and Living Alone

**DOI:** 10.1093/geroni/igaf122.370

**Published:** 2025-12-31

**Authors:** Jennifer Lay, Yuen Wan Ho, Dwight Tse, Helene Fung, Da Jiang

**Affiliations:** University of Exeter, Exeter, England, United Kingdom; Lingnan University, Hong Kong, Hong Kong, China (People’s Republic); University of Strathclyde, Glasgow, Scotland, United Kingdom; The Chinese University of Hong Kong, Hong Kong, China (People’s Republic); The Education University of Hong Kong, Hong Kong, China (People’s Republic)

## Abstract

Recalling past affective experiences is key to guiding behaviour – including solitary activities – in ways that maximise wellbeing. However, we do not always accurately recall such experiences. Previous research suggests older (vs. younger) adults show a positivity effect in memory, depending on whether the content is culturally meaningful. It is unclear, however, whether this age-related positivity effect applies to recalling time in solitude (no social contact), which becomes increasingly prevalent in older adulthood. An age-stratified sample of adults aged 18-81 (M age = 40.8 years, 60.8% female) in the United Kingdom (N = 214) and Hong Kong (N = 220) provided a total of 13,531 reports of their current affective experiences and social situations over a 7-day period (5 times/day) using a smartphone app. Participants then recalled how they felt over the 7-day period at times when they were in solitude. Models controlling for peak and recent affect, overall time in solitude, independent/interdependent self-construal, and demographic factors showed that whereas younger adults tended to retrospectively overestimate how lonely they felt in solitude, older adults tended to underestimate their loneliness in solitude. Moreover, whereas UK older adults tended to retrospectively overestimate their happiness in solitude, HK older adults and those who were living alone tended to underestimate happiness in solitude. Findings suggest individuals misremember their solitude experiences in ways that align with age-normative and cultural attitudes to solitude. Additionally, there is an age-related positivity effect which is shaped by culture. We discuss implications for loneliness and wellbeing in older adulthood.

